# Timing of Administration: For Commonly-Prescribed Medicines in Australia

**DOI:** 10.3390/pharmaceutics8020013

**Published:** 2016-04-15

**Authors:** Gagandeep Kaur, Craig L. Phillips, Keith Wong, Andrew J. McLachlan, Bandana Saini

**Affiliations:** 1 Faculty of Pharmacy, The University of Sydney, Camperdown NSW 2006, Australia; andrew.mclachlan@sydney.edu.au (A.J.M.); bandana.saini@sydney.edu.au (B.S.); 2 Woolcock Institute of Medical Research, University of Sydney, Glebe, NSW 2037, Australia; craig.phillips@sydney.edu.au (C.L.P.); keith.wong@sydney.edu.au (K.W.); 3 Department of Respiratory & Sleep Medicine, Royal North Shore Hospital, Sydney NSW 2065, Australia; 4 Department of Respiratory & Sleep Medicine, Royal Prince Alfred Hospital, Camperdown NSW 2050, Australia; 5 Centre for Education and Research on Ageing, Concord Hospital, Concord, NSW 2137, Australia

**Keywords:** chronotherapy, circadian rhythm, medicines, statins, antihypertensives, proton pump inhibitors, timing of drug administration, Australia

## Abstract

Chronotherapy involves the administration of medication in coordination with the body’s circadian rhythms to maximise therapeutic effectiveness and minimise/avoid adverse effects. The aim of this study is to investigate the “time of administration” recommendations on chronotherapy for commonly-prescribed medicines in Australia. This study also aimed to explore the quality of information on the timing of administration presented in drug information sources, such as consumer medicine information (CMI) and approved product information (PI). Databases were searched for original research studies reporting on the impact of “time of administration” of the 30 most commonly-prescribed medicines in Australia for 2014. Further, time of administration recommendations from drug information sources were compared to the evidence from chronotherapy trials. Our search revealed 27 research studies, matching the inclusion and exclusion criteria. In 56% (*n* = 15) of the research studies, the therapeutic effect of the medicine varied with the time of administration, *i.e.*, supported chronotherapy. For some medicines (e.g., simvastatin), circadian-based optimal administration time was evident in the information sources. Overall, dedicated studies on the timing of administration of medicines are sparse, and more studies are required. As it stands, information provision to consumers and health professionals about the optimal “time” to take medications lags behind emerging evidence.

## 1. Introduction

The “*time*” of administration of medication is valuable information to consider during patient counselling and is a typical query by patients especially when filling a prescription for the first time [1]. Various therapeutic management support systems and novel technologies have been developed to assist health professionals, including clinical pharmacists, in ensuring the rational and safe use of medicines. One such “tool” in the armoury of clinical pharmacists is to exploit the knowledge of biological rhythms in humans. A human biological rhythm with a period of around 24 h is known as circadian rhythm. Circadian rhythms are endogenous in nature and are known to persist under free-running conditions [2,3,4].

**Figure 1:**
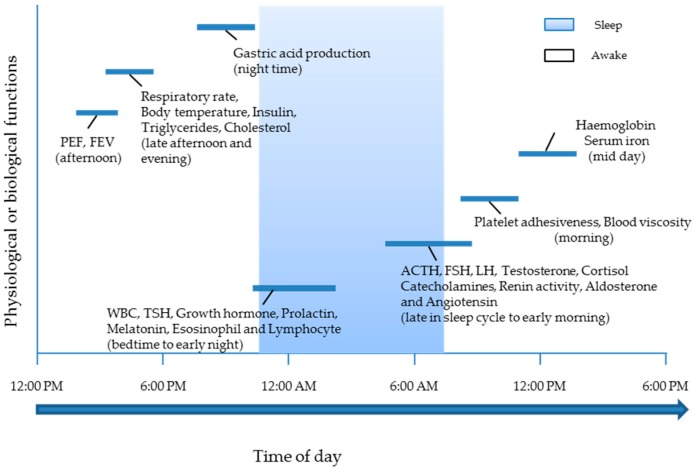
Time of day when physiological or biochemical functions are at peak [16,17]. PEF, peak expiratory flow rate; FEV, forced expiratory volume; WBC, white blood count; TSH, thyroid stimulating hormone; ACTH, adrenocortical tropic hormone; FSH, follicle stimulating hormone; LH, luteinizing hormone.

**Figure 2:**
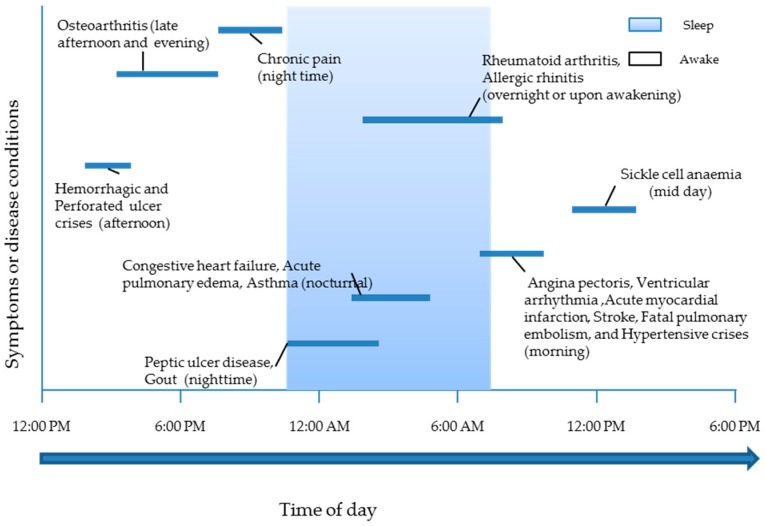
Time of day when symptoms or events of diseases are most frequent [16,17].

Circadian rhythms are governed by a network of hierarchical master clocks present at various locations in the brain and peripheral tissues, such as liver [4,5]. Of these clocks, the most important are the paired suprachiasmatic nuclei (SCN) located in the hypothalamus, which coordinate the multitude of these other clock networks via endocrine and neural signals [6]. Specialised cells in the retina convey the onset and offset of daylight to the SCN and pineal gland [5] and form one of the external environmental signals (zeitgebers), which keep the circadian rhythms in pace. This gives rise to rhythmic variations in the physiological status of the body’s systems and even influences the susceptibility of human beings to morbid and mortality events. For example, the incidence of myocardial infarction mostly occurs between 6:00 a.m. and 12:00 noon [7]. Diseases, such as asthma [8], allergic rhinitis [9], cancer [10], cardiovascular diseases [11], osteoarthritis [12] and peptic ulcers [13], exhibit circadian rhythms in the occurrence or intensity of symptoms [14,15]. Figure 1 and Figure 2 highlight further examples.

Pharmacists can exploit this knowledge of circadian rhythms by employing chronotherapy principles, which involve the timing of the administration of dosage forms, conventional or special, to deliver medication in coordination with the body’s circadian rhythms to maximise therapeutic effectiveness whilst also minimising or avoiding adverse effects [18]. Chronotherapy requires an understanding of circadian patterns in the intensity of disease symptoms and rhythmic patterns in the human body’s handling of medicines, which determine the therapeutic and adverse effects of a medicine [19]. Studies have documented circadian variability in pharmacokinetics and pharmacodynamics for medicines, according to their circadian time of ingestion, influencing the therapeutic and adverse effects [20,21]. Chronopharmacokinetics is the field of study that explores circadian variability in the absorption, distribution, metabolism and elimination of medicines [22,23]. Theophylline, for example, was one of the first medicines for which circadian variation in pharmacokinetic parameters was demonstrated with higher *C*_max_ and shorter *T*_max_ after administration in the morning when compared to evening administration [24,25,26,27]. Circadian rhythmicity at the cellular and sub-cellular level can also give rise to significant differences in the pharmacodynamics of medicines depending on the time of administration [28]. Chronopharmacodynamics is the field of study that explores circadian variability in the mode of action of the medicine [3] and matches administration to achieve optimal pharmacodynamic response in the circadian cycle for a particular medicine. For example, rhythmicity in receptor activity has been shown for the interferon-alpha (IFN-α) receptor. Thus, the antiviral effect of IFN-α can be altered via its administration time [29].

The first use of chronotherapy in practice was introduced in the 1960s when morning ingestion of corticosteroid medication was adopted to reduce its adverse effects. This was achieved by timing ingestion of a conventional immediate release tablet to occur in synchrony with the circadian peak of cortisol release by the adrenal cortex [17,30]. Since that time, there has been an increased emphasis on circadian rhythms and chronotherapy studies. It is important to note that there have been multiple published chronotherapy studies covering a wide range of medicines and disease conditions. These include cardiovascular drugs (angiotensin II receptor blockers, beta-blockers, calcium channel blockers, ACE-inhibitors, organic nitrates, cardiac glycosides) [4,31,32,33,34], anti-asthmatics (theophylline, beta-agonists, leukotriene antagonists, glucocorticoids) [8], anticancer agents (5-flurouracil, methotrexate, 6-mercaptopurine, platinum analogues, doxorubicin) [35], opioids, NSAIDs and local anaesthetics [36].

International guidelines have also started taking into consideration the circadian rhythms in the manifestation and intensity of disease conditions. For example, ambulatory blood pressure monitoring (ABPM) is recommended in the USA by the Agency for Healthcare Research and Quality, U.S. Department of Health and Human Services [37]. In Canada and the United Kingdom, ABPM is also recommended as the preferred method for the diagnosis of arterial hypertension. Similarly, the Ambulatory Blood Pressure Monitoring Working Group (2011) in Australia emphasises the potential benefits of normalisation of the circadian blood pressure variability in hypertension, particularly by using chronotherapy [38]. Chronotherapies have been used in the United States, Europe and Asia [17]; for example, once-daily evening administration of specially-formulated theophylline tablets in the treatment of chronic obstructive pulmonary disease and conventional H2-receptor antagonists with recommended evening administration for peptic ulcer disease [16,17,39].

The Pharmaceutical Benefits Scheme (PBS) provides Australian residents with subsidised access to prescription medicines in a manner that is affordable, reliable and timely [40]. Because of this system, most prescription medicine sales are recorded by dispensary systems, and these data are made available online through the PBS website. The exceptions in this data capture would be non-subsidised prescription medications and over the counter medications [40]. The 30 most commonly-prescribed medicines through PBS constitute 46% of the total PBS volume of prescriptions dispensed and 27% of the total AUD 7.3 billion government healthcare expenditure on providing subsidised medicines in Australia [41]. Most of these 30 commonly-prescribed medicines (*n* = 27) have been on the PBS list consistently over the last five years (Table 1). In order to reduce medication costs, efficient ways to improve their effectiveness, such as adherence support, prevention of stockpiling and medication reviews, are commonly used. Chronotherapy may be another method of improving medication effectiveness by timing ingestion to occur at circadian times where drug effects can be maximised and/or adverse effects minimised. Reviewing whether this option applies to the 30 most commonly-prescribed medicines may have a broader implication for healthcare in Australia and even across other countries where chronic illness and medication use profiles are similar.

### Aim

Firstly, this study aimed to investigate the “time of administration” recommendations on chronotherapy for commonly-prescribed medicines in Australia. Secondly, given that where evidence exists, it needs to be disseminated to patients and professionals, the study also aimed to gauge how well the information on the timing of administration is presented in drug information sources, such as Consumer Medicine Information (CMI) and Australian Approved Product Information (PI) materials.

## 2. Experimental Section

### 2.1. Operationalising Commonly-Prescribed Medicines

The 30 most commonly-prescribed medicines (by volume) in the year July 2013–June 2014 were selected from the publically available PBS data [41]. This category of medicines is likely to be similar in most developed countries [42].

### 2.2. Literature Search and Analysis

Embase and Medline were searched for original research studies reporting on the impact of time of administration for the 30 most commonly-prescribed medicines as reported by PBS, Australia, for 2014. For each medicine, the generic name of the medicine and the terms “circadian rhythms”, “drug chronotherapy”, “chronopharmacology”, “chronopharmacokinetics”, “chronopharmacodynamics”, “time-dependent effect”, “drug administration schedules”, “morning *vs.* evening”, “morning *vs.* bedtime” and “morning *vs.* night-time” were used for searching the literature using “AND” and “OR” boolean operands. For each of the 30 iterations of the search above, inclusion criteria for articles were original research, human subjects and study presented in English language. The exclusion criteria for the selection of studies were that the following research studies would be excluded: research performed with children or pregnant women, healthy subjects, non-comparative studies (e.g., where the trial had drug administration at one set time) and studies conducted with a small size (10 or less subjects). Duplicate articles were then removed using a bibliographic tool, Endnote X7 (Thomson Reuters, United States). Among the included studies were randomised controlled trials, comparative trials (drug administration done at more than one time point), combination trials (more than one drug combination) and with patients. The selected studies were assessed for chronotherapy recommendations, *i.e.*, whether the study results could clearly answer the question: “Is there a specific circadian ‘time or time range’ of medication administration that can result in better effect for the given medicine?”

### 2.3. Analysis of Consumer Medicine Information and Australian Approved Product Information

In Australia, the Therapeutic Goods Administration (TGA) regulation requires approved PI to be provided by the pharmaceutical companies to assist doctors, pharmacists and other health professionals in prescribing and dispensing medicines. The approved PI provides objective information about the quality, safety and effectiveness of the medicine. Similarly, the TGA requires that CMI provide the safe and efficient use of the medicine to the patients in English, easy to understand (non-technical) language and in a standardised format [43,44].

To assess how well a time of administration recommendation for the 30 most commonly-prescribed medicines was readily made available to health professionals and consumers, the CMIs and PIs for these medicines were obtained and their content analysed [45]. The text in these documents was explored for specific information or advice on a specific time of medicine administration under the “*Dosage and Administration*” heading in the full PI and under the “*Instructions on when to take the medication*” in the CMI for all 30 medicines. If the material under these headings made clear reference to the ideal “time” when the medicine should be used and this approved time was found to be consistent with evidence from chronotherapy trials, the document (CMI or PI) was scored as positive.

## 3. Results and Discussion

“Time of administration” English language studies have been reported for 40% (*n* = 12) of the 30 most commonly-prescribed medicines. These included atorvastatin, simvastatin, perindopril, ramipril, irbesartan, telmisartan, candesartan, amlodipine, atenolol, rabeprazole, omeprazole and tiotropium. For these 12 medicines, the search revealed 27 research studies matching the inclusion and exclusion criteria. Out of the 27 research studies, 56% (*n* = 15) indicated that the therapeutic effect of the medicine varied with the time of administration of medication, *i.e.*, supported chronotherapy. These studies included mostly statins, antihypertensives and a proton pump inhibitor.

Previous studies have demonstrated a reproducible relationship between low-density lipoprotein-cholesterol (LDL-C) concentration achieved and absolute cardiovascular risk [46]. Hence, for statins, the assessment for chronotherapy was based on the ability to lower the LDL-C concentrations. Studies have demonstrated that in hypertensive patients, a reduced blood pressure (BP) drop during sleep is associated with increased cardiac events [47]. Hence, antihypertensives were assessed based on their ability to control nocturnal BP. Based on the findings of these morning-evening administration time studies, morning administration is suggested for amlodipine, evening/bedtime administration for ramipril, candesartan, telmisartan, amlodipine/hydrochlorothiazide, amlodipine/olmesartan and amlodipine/valsartan, night-time administration for perindopril and evening administration for atorvastatin, simvastatin, amlodipine/valsartan and rabeprazole. For the remaining 44% (*n* = 12) of the studies, the therapeutic effect of the medicine did not vary with the time of medicine administration.

### 3.1. Statins

Of the 27 studies reviewed, nine studies evaluated the chronotherapy of statins (atorvastatin and simvastatin) (Table 2). Five out of nine studies supported the administration time dependency of the lipid lowering effect for statin use [48,49,50,51,52]. In the case of atorvastatin, a prospective randomised trial conducted with 152 people with hyperlipidaemia undergoing their first elective percutaneous coronary intervention demonstrated statistically-significant reductions in lipid concentrations for evening administration. The patients were randomised to receive their atorvastatin dose (40 mg/day for the first month and 10 mg/day ongoing regimen) either in the morning (Group I, *n* = 73) or in the evening (Group II, *n* = 79). Lipid profiles were compared between both the groups at baseline and six months of therapy. After six months, LDL-C concentration decreased by 5 mg/dL, and total cholesterol (TC) concentration decreased by 4 mg/dL in Group II, as compared to Group I (both *p* < 0.05) [48]. However, a study conducted by Plakogiannis *et al.* found that atorvastatin (40 mg) showed no significant difference in lipid lowering effect between morning and evening administration [53]. The study lacked a randomised design, and all of the subjects were hyperlipidaemic males.

For simvastatin, the literature search identified seven studies, and most reported a chronotherapeutic benefit with evening administration. For example, a placebo-controlled, double-blind study compared the effectiveness of morning *vs.* evening administration of simvastatin for two different doses (2.5 and 5 mg) [49]. Hyperlipidaemic patients (*n* = 172) were randomised into five groups. After 12 weeks of treatment, the percent decrease in LDL-C concentrations when compared to baseline was greater in patients taking simvastatin in the evening than in the morning (−22.2% *vs.* −15.2% for simvastatin 2.5 mg and −28.5% *vs.* −19.3% for simvastatin 5 mg). The reduction in LDL-cholesterol concentration for evening administration was statistically significant for the 5 mg dose when compared to morning administration. In another study, Wallance *et al.* reported a significant increase of 10% in the LDL-C concentration from baseline (95% CI 0.06–0.44; *p* = 0.012) in 57 hyperlipidemic patients switching simvastatin from evening to morning administration [50]. Similar results were obtained from other studies on simvastatin, including patients with coronary artery disease [51] and dyslipidaemia [52]. Collectively, these studies indicate that simvastatin has greater LDL-C concentration percentage reduction when taken in the evening. However, a trial comparing morning *vs.* evening administration for a controlled release (CR) simvastatin demonstrated no statistically-significant differences between morning and evening treatment [54]. In fact, recent evidence has highlighted that morning administration of CR simvastatin is equivalent to that of evening administration of immediate-release (IR) simvastatin for lowering LDL-C, TC and TG (triglyceride) concentrations [55]. Similarly, simvastatin in combination with ezetimibe also did not show any significant differences in LDL-C concentration reduction, after morning (46%, *p* < 0.001 from baseline) or evening administration (48%, *p* < 0.001 from baseline) [56].

The FDA approved evening administration for simvastatin. It is based on the rationale of the short half-life (2–3 h) of simvastatin [57]. Atorvastatin, its active metabolite and rosuvastatin all have long half-lives (14, 20 and 30 h, respectively), and the FDA approved “any time” administration for these medicines [57]. Given this definitive direction, the analyses of CMI and PI for “administration time” evidence for simvastatin was scored as positive, as it provided explicit advice that “evening” is the preferred time of administration, and that is consistent with the evidence from the reviewed studies for simvastatin. The CMI and PI approved “any time of the day” for administration of atorvastatin and rosuvastatin.

### 3.2. Antihypertensive Medications

Of the twenty-seven studies reviewed, fifteen investigated the chronotherapy of antihypertensive medications. These studies include perindopril, ramipril, irbesartan, telmisartan, candesartan, atenolol and amlodipine (Table 3). Nine of these fifteen studies demonstrated different effects on blood pressure control depending on the time of administration [58,59,60,61,62,63,64,65,66].

Supporting chronotherapy for antihypertensives was a randomised crossover study reported by Morgan *et al*. This study demonstrated that the duration of the blood pressure lowering effect of perindopril (4 mg) when administered to 18 hypertensive men was 24 h when given in the morning at 9 a.m., whereas it was only 12–14 h when administered at 9 p.m. However, the magnitude of reduction in BP while sleeping was greater with night-time administration than morning (−11.7/−7.2 mmHg *vs.* −8.2/−5.2 mmHg, *p* < 0.05) [58]. In another randomised crossover trial, 2.5 mg ramipril was administered to 33 hypertensive patients, and this effectively reduced daytime blood pressure (−6.7/−5.7 mmHg) after morning administration and decreased nighttime blood pressure (−4.3/−3.0 mmHg) when administered in the evening. However, the 24-h blood pressure control lasted slightly longer after morning administration compared to an evening dose (16 h *vs.* 15 h) [67]. The small dose of ramipril (2.5 mg) might have failed to cover the entire 24-h control of blood pressure. In yet another study of ramipril (5 mg) with 115 essential hypertensive subjects, bedtime administration significantly reduced BP during sleep in comparison to the morning administration (−13.3/−11.5 mmHg *vs.* −4.5/−4.1 mmHg, *p* < 0.001) [59].

Similar results were found in studies analysing the effects of angiotensin-II receptor blockers (ARB). For example, bedtime administration of telmisartan (80 mg) to 215 participants with essential hypertension resulted in significant decreases in BP during sleep when compared to morning administration (−13.8/−9.7 mmHg *vs.* −8.3/−6.4 mmHg, *p* < 0.001) [60]. Similarly, bedtime administration of candesartan (4 mg and 8 mg) increased baroreflex sensitivity (confers renal protection) in patients with hypertension (*n* = 109) more than a morning dose [62]. In an another randomised crossover study conducted by Pechère-Bertschi *et al.*, irbesartan (100 mg) administered to 10 uncomplicated essential hypertension subjects demonstrated a decrease of systolic asleep BP by 7.4 mmHg and 4.2 mmHg for evening and morning administration, respectively, after six weeks of treatment. However, the difference was not statistically significant between the administration times [61]. Similarly, clinical studies involving amlodipine demonstrated a reduction in BP throughout 24 h whether ingested in the morning or evening [68,69]. However, in a prospective, 12-week, double-blind, randomised, crossover study of mild-to-moderate essential hypertension patients (*n* = 60), the nocturnal BP fall was greater with morning than with evening administration (−9.8/−7.4 *vs.* −6.7/−5.4 mmHg, *p* < 0.01/0.05) [70]. The combination of amlodipine/hydrochlorothiazide [63], amlodipine/olmesartan [64] and amlodipine/valsartan [65,66] showed higher effectiveness for bedtime than morning administration. However, in a study conducted by Asmar *et al.*, the authors reported not finding any significant differences between morning and evening administration of amlodipine/valsartan [71].

In the case of atenolol, a chronopharmacokinetics trial investigated the effect of a 50-mg dose in 13 hypertensive patients and found no significant differences in C_max_ and T_max_ for morning *vs.* night-time administration.

The analyses of CMI and PI for current administration time evidence for antihypertensives revealed that administration time is available only for perindopril. CMIs and PIs approved “morning” administration for perindopril, whereas the literature evidence suggests bedtime administration. The evidence from the literature is consistent with the American Diabetes Association recommendation of bedtime administration of at least one antihypertensive medicine [73].

### 3.3. Proton Pump Inhibitors

Of the twenty-seven studies reviewed, two studies evaluated administration time dependency for proton pump inhibitors (PPIs) (Table 4). Both studies had small sample sizes and did not report whether the studies were powered. In the case of rabeprazole 20 mg, evening administration in gastroesophageal reflux disease patients (*n* = 20) normalised more effectively the total oesophageal acid exposure and provided significantly better control of nocturnal gastro-oesophageal reflux disease [74]. In the case of omeprazole 40 mg, the mean gastric pH during daytime was higher after morning administration than after evening administration (0.72 ± 0.91 pH, *p* < 0.0 1); whereas, the mean gastric pH during the nighttime was greater after evening administration than after morning administration (0.64 ± 0.83 pH, *p* = 0.02). This suggests that morning administration of omeprazole is preferable for patients with reflux resulting from physical activity, whereas patient with nocturnal reflux prefer evening administration [75].

We did not find any recommendation on the time of administration in either the CMIs or PIs for PPIs. It was interesting to note that the FDA approved rabeprazole 20 mg to be taken after a morning meal [76].

### 3.4. Anticholinergics

Only one study evaluated the chronotherapy basis for anticholinergic agents (Table 5). In a double-blind, randomised, placebo-controlled study in stable chronic obstructive pulmonary disease patients (*n* = 121) treated with inhaled tiotropium once daily in the morning or the evening, no significant differences in the morning *vs.* evening administration were shown [77].

### 3.5. Discussion

This study has examined the availability, currency and accuracy of the information available around the preferred “time” of administration for commonly-prescribed medicines in Australia. The study revealed that the dissemination of “time of administration” recommendation information into drug information sources, like CMIs and PIs, for the 30 most commonly-prescribed medicines in Australia lags behind available evidence. In clinical trials where medicine administration at two different circadian times was compared for effectiveness, results supported chronotherapy for only eight medicines in the PBS list. These included statins, antihypertensives and PPIs. It is important to note that these eight medicines where data have supported potential for chronotherapy represent 13% of all of the PBS prescriptions (and 7% of the government’s PBS cost) for the year 2014 in Australia. Clearly, improved efficacy of these medicines could lead to economic efficiencies, not only for the PBS in Australia, but national health schemes or health insurance agencies globally.

Statins have become the first-line therapy for reducing the risk of cardiovascular disease (CVD) mortality and morbidity in patients with a suboptimal lipid profile, with or without other risk factors [78]. It is known that the rate of cholesterol biosynthesis is physiologically highest after midnight and lowest during the morning and early afternoon [79]. Diurnal changes in the activity of hydroxymethylglutaryl coenzyme A (HMG-CoA) reductase causes this circadian variation [80]. The circadian variation in the effect of simvastatin with evening administration demonstrating better effects can be clearly attributed to circadian variation in cholesterol synthesis. Unlike simvastatin, other statins have longer half-lives and, so, may have sufficient plasma concentrations through 24 h, making their effect less reliant on administration time. Furthermore, the statin trials we included all had different durations of follow up, *i.e.*, at 2, 4, 8, 12 or 52 weeks. It may be noted that although the lipid lowering effect of statins is evident within days, most clinical guidelines do suggest assessment every six weeks until the target goal has been achieved [81,82].

Similarly, the cardiovascular system displays circadian variability in function, in relation to both hormonal and biochemical regulation [83]. Normotensive and primary hypertensive patients display a nighttime fall in their blood pressure (dippers); whereas patients with secondary hypertension (where the cause may be renal disease or gestational diabetes) display an increase in nocturnal blood pressure (non-dippers) [84]. Prospective observational studies have concluded that nocturnal blood pressure is a better predictor of a worse prognosis in comparison with 24-h blood pressure or daytime blood pressure [85,86,87]. Several studies, among them the Heart Outcomes Prevention Evaluation (HOPE) [88,89] and Monitorización Ambulatoria de la Presión Arterial y Eventos Cardiovasculares (*i.e.*, Ambulatory Blood Pressure Monitoring and Cardiovascular Events) (MAPEC) [90] trials, have reported that a bedtime treatment strategy of one or more conventional hypertension medications affords better 24-h BP control and greater protection against cardio vascular incidents than the traditional morning time strategy. In patients where the nocturnal blood pressure does not dip, nighttime administration of antihypertensive may assist by promoting dipping. In this context, the circadian variation in the renin–angiotensin–aldosterone system and its activation during nocturnal sleep has been hypothesised to explain the BP lowering effects of bedtime ingestion of angiotensin-converting enzyme (ACE) inhibitor and ARB medications [32]. Other circadian drug disposition effects (for example ACE inhibitors, which are prodrugs) may be explained by circadian variation in hepatic de-esterification. During sleep, hepatic flow is reduced, which may decrease the activation of ACE inhibitors, resulting in a delayed peak effect [91]. Furthermore, circadian variation in glomerular filtration rate, urinary pH, renal blood flow and tubular reabsorption are well known and can alter the medicine and metabolite elimination [92,93].

It is also important to note that the blood pressure lowering effect after evening/bedtime administration may result in the risk of systemic hypotension, thereby increasing the risk to patients with small vessel disease from injury to the eye, brain and other organs of the body due to inadequate blood perfusion [39]. Therefore, greater care needs to be taken to avoid adverse events, preferably by titration of the dose after conducting 24-hour or longer ABPM to ensure therapeutic efficacy and safety.

In the case of the gastrointestinal system, circadian rhythms may influence acid secretion and gastric motor activity. For example, gastric acid secretion is highest during the night [94,95]. Suppression of nocturnal acid is an important factor in duodenal ulcer healing [96]. In this context, it has been suggested that the optimal time for administration of medicine may depend on the type of PPI, clinical symptoms and patient preferences [80].

It is surprising to note that most of the studies evaluating the impact of chronotherapy were either small; with a limited number of subjects, single-centred or uncontrolled or non-blinded. Many of the studies did not take into account variations in the sleep-wake routines of participants, *i.e.*, patients with a normal routine (diurnal activity and nighttime sleep) or night/shift workers, which can alter the circadian physiology in these cases. Most of the studies failed to define the timing of medicine administration in the Methods section. For example, merely recording morning and evening administration according to day and night may not be sufficient, as definitions of the morning and evening may be different for different patients. For these chronotherapy studies, the time of drug administration may be best defined as hours before or after actual sleep. For example the studies performed by Hermida *et al*. routinely rely on wrist actigraphy and/or patient diaries to accurately determine the onset and offset of nighttime sleep. Furthermore, important to note is the fact that most of the reviewed studies compared morning *vs.* bedtime/evening administration, perhaps out of concern for patient compliance/adherence issues. It is unknown if the choice of other times, such at midday or afternoon, would reveal greater therapeutic effects and outcomes. Given this limitation, it may be stated that the true assessment of the best time to administer a medication in most cases has not been appropriately and comprehensively explored. Hence, knowledge of the patients’ sleep and the circadian cycle is essential for designing chronotherapy trials [17].

Guidelines, such as the American Diabetes Association, recommended that at least one antihypertensive drug be given at bedtime [73]. However, bedtime administration has not been specified in CMIs and PIs for antihypertensive medications. The information within the Australian approved PI and CMI is provided by pharmaceutical companies or drug manufacturers to be reviewed and approved by medicine regulatory agencies, such as TGA [97], and are usually derived from clinical studies involving a single administration time. These studies are mostly based on homeostatic design, *i.e.*, they entail a single time of day of dosing with pharmacokinetics and pharmacodynamics assessments performed during daytime at the convenience of the diurnally-active investigators and diurnally-active study subjects [39]. Chronotherapy studies are not requested for drug evaluations by the regulatory authorities [98]. Most pharmaceutical companies may not be conducting studies on possible circadian and other rhythmic influences on drug pharmacokinetics and therapeutic effects, particularly for medicines already marketed [39]. Thus, there could be gaps between the published literature *vs.* the approved “time of administration” recommendation of medications. Preliminary screening of new drugs for their chronotherapeutic potential is a way of enhancing the research and development in pharmaceutical industries [98]. Elucidating the role of the molecular clock and circadian molecular pathways could provide a major advance in treatment options for patients. For example, targeting a circadian molecular pathway at a predictable time point, when the pathway is unregulated, will result in more efficacious therapies, active for shorter periods of time, with fewer side effects. For medicines where chronotherapy-based formulations exist, vigorous dissemination of this concept in scientific and industry circles could encourage research and improve formulation aspects [99].

This study highlighted that the timing of drug administration was available only for simvastatin and perindopril in their respective CMIs and PIs. Given the lack of dissemination about chronotherapy in regularly-used sources of information by health professionals, it may be difficult for healthcare professionals to counsel patients on the appropriate time of administration, such as “before or after a meal” or in a non-specific way of “morning or evening administration”. The instructions may not always be about patients’ routine awakening or bedtimes.

Knowledge about circadian influence on the therapeutic effect of medicine should be used during patient counselling, especially if the medicine is being taken for the first time. A recent research program in Germany developed a database containing optimum timing of drug administration that could help to optimise treatment regimens in clinical practice [100]. Healthcare professionals should benefit from readily available knowledge about which medicine has evidence supporting chronotherapy so that patient outcomes can be improved. Integrating chronotherapy evidence with information on medication is an important first step in promoting a change in prescribing practices. Similarly, providing pre-registration health professionals with a current understanding of chronotherapy through health curricula may be a necessary way to bridge this evidence-practice translation [101]. A recent study has developed a chronotherapy-based educational training program for pharmacy graduates. The study highlights that the role of an up-to-date, evidence-based educational program on chronotherapy can improve knowledge and enhance positive attitudes for pharmacist roles in clinical practice [102].

### 3.6. Limitations

Our study has some limitations worth mentioning. Firstly, we only considered the 30 most common medicines in the year 2014, in Australia. It is beyond the scope of this article to provide the data supporting chronotherapy information that was not included in the 30 most common medicines. Therefore, we might fail to retrieve review publications about chronotherapy of other classes of medication, such as anti-cancer medications. Secondly, in our literature review, we excluded studies that were dealing with the stability of a medicine rather than the effect of administration at different circadian times. Thirdly, we limited our search to two databases, studies published in English only and those published in peer-reviewed journals as original research manuscripts; this might have excluded studies published, for example, as conference proceedings or those published in languages other than English. Fourthly, many studies included in the review section of our research did not have large numbers of patients; this is the case, in fact, in many other chronotherapy studies [57,100,103]. Our intent was not to conduct a systematic review or meta-analysis of the collated findings from studies. In any case, this exercise would be hindered by the fact that the number of studies for any given medicine is small and probably, per medicine, not enough to conduct a systematic review. Finally, although we made reference to whether the information is made available to patients in the CMI, and PI documents, both healthcare professionals and their patients may have access to a wider range of information materials (e.g., the Internet) that they may act upon.

## 4. Conclusions

For the 30 most commonly-prescribed medicines in Australia, our review highlighted that there is some evidence regarding the optimal time of administration for ACE-inhibitors, angiotensin receptor blockers and statins. There are studies for other medicines where the evidence is inconclusive. For medicines such as statins, where there is clear evidence of increased cholesterol biosynthesis nocturnally, evening administration is clearly recommended in the literature and the CMI and PI, particularly for the medicines with a shorter half-life (simvastatin). For those studies where evidence only showed trends, references to “time” were not included in the CMI and PI. Thus, dedicated studies on the timing of administration of medicines are sparse, and more studies are required. As it stands, information provision to patients and health professionals about the “best circadian time” to take medications lags behind emerging evidence.

## Figures and Tables

**Table 1:** Top 30 commonly-prescribed medicines by generic name (June 2014).

Rank	Drug	Rank	Drug
1	Atorvastatin	16	Amoxicillin
2	Rosuvastatin	17	Ramipril
3	Esomeprazole	18	Paracetamol + Codeine
4	Paracetamol	19	Amlodipine
5	Pantoprazole	20	Irbesartan + Hydrochlorothiazide
6	Perindopril	21	Venlafaxine
7	Metformin	22	Clopidogrel
8	Fluticasone + Salmeterol	23	Omeprazole
9	Irbesartan	24	Amoxicillin + Clavulanic Acid
10	Simvastatin	25	Candesartan
11	Salbutamol	26	Rabeprazole
12	Atenolol	27	Telmisartan ^†^
13	Cephalexin	28	Tiotropium
14	Oxycodone	29	Tramadol ^†^
15	Warfarin	30	Desvenlafaxine *

^†^ Among the top 30 for 3 out of the last 5 years, * new in 2014, the rest consistently in top 30 for the last 5 years.

**Table 2:** Evidence supporting chronotherapy of statins.

Study	Participants (Sample Size) Age in Years	Medicine (Dose)	Study Design (Study Duration)	Suggested Time
Ozaydin *et al.* [48]	Hyperlipidemic patients (*n* = 152, 118 male) Age: 59 ± 5	Atorvastatin (40 mg followed by 10 mg)	Prospective randomised study Morning/evening (12 months)	Evening
Plakogiannis *et al*. [53]	Hyperlipidemic patients taking Atorvastatin 40 mg (*n* = 64 males) Age: 58.5 ± 7.8 (evening group), 57.8 ± 7.8 (morning group)	Atorvastatin (40 mg)	Comparative study, Morning (before noon)/evening (after 1800 h and before midnight) (4 weeks)	Morning/evening
Saito *et al.* [49]	Hyperlipidemic patients (*n* = 150, 33 males) Age: 18–65	Simvastatin (2.5 mg and 5 mg)	Double-blind, randomised, placebo-controlled study Morning/evening (12 weeks)	Evening
Wallace *et al.* [50]	Hyperlipidemic patients (*n* = 57, 27 males) Age: 44–82	Simvastatin (10 mg, 20 mg)	Randomised study Morning/evening (8 weeks)	Evening
Lund *et al.* [51]	Coronary Artery Disease patients (*n* = 25, 18 males), Age: 66 ± 11	Simvastatin (10, 20 and 40 mg)	Randomised crossover study Morning/evening (12 weeks)	Evening
Tharanvanij *et al.* [52]	Dyslipidaemia subjects (*n* = 52) Age: 18–70	Simvastatin (10 mg)	Randomised, double-blind, controlled study, Morning (0600–1000 h)/evening (1900–2200 h) (12 weeks)	Evening
Kim *et al.* [54]	Dyslipidaemia Korean subjects (*n* = 132, 55 males) Age: 58.7± 8.3 (MG) 58.5 ± 9.5 (EG)	Simvastatin controlled release (20 mg)	Prospective, randomised, double-blind, placebo controlled, multicentre study Morning/evening (8 weeks)	Morning/evening
Yong *et al.* [55]	Dyslipidaemia patients with chronic kidney disease (*n* = 122, 57 males), Age: 20–75	Simvastatin Controlled release (CR) and immediate-release (IR) (20 mg)	Prospective, randomised, multicentre, double-blind study Morning (CR)/evening (IR) (8 weeks)	Morning/evening
Yoon *et al.* [56]	Primary hypercholesterolemia patients (*n* = 145, 101 males) Age: 18+	Ezetimibe/simvastatin (10 mg/20 mg)	Multicentre, open label, randomised, 2-sequence, 2 period crossover study Morning/evening (6 weeks)	Morning/evening

MG: Morning group; EG: Evening group.

**Table 3:** Evidence supporting the chronotherapy of antihypertensive agents.

Reference	Participants (Sample Size) Age in Years	Medicine (Dose)	Study Design (Study Duration)	Suggested Time
Morgan *et al.* [58]	Essential hypertensive patients (*n* = 18 males) Age: 33–85	Perindopril (4 mg)	Randomised crossover study, Morning (0900 h)/nighttime (2100 h) (4 weeks)	Nighttime
Hermida *et al.* [59]	Uncomplicated essential hypertensive patients (*n* = 115, 52 males) Age: 46.7 ± 11.2	Ramipril (5 mg)	Multicentre, PROBE, parallel group study Awakening/bedtime (6 weeks)	Bedtime
Myburgh *et al.* [67]	Mild to moderate essential hypertensive patients (*n* = 39, 35 males) Age—24–73	Ramipril (2.5 mg)	Open, randomised, crossover study Morning (0800 h)/Evening (2000 h) (4 weeks)	Morning/evening
Hermida *et al.* [60]	Grade 1 or 2 essential hypertensive patients (*n* = 215, 114 males) Age: 46.4 ± 12.0	Telmisartan (80 mg)	PROBE, parallel group study, Morning/bedtime (12 weeks)	Bedtime
Pechere-Bertschi *et al.* [61]	Mild to moderate essential hypertensive patients (*n* = 10) Age: 35–70	Irbesartan (100 mg)	Randomised, double-blind, double-dummy, crossover study Morning/evening (12 weeks)	Morning/evening
Eguchi *et al.* [62]	Patients having at least one antihypertensive medicine or been unmedicated (*n* = 109, 70 males) Age: 58.3 ± 11.3	Candesartan (4 mg, 8 mg)	Randomised study Morning/bedtime (6 months)	Bedtime
Nold *et al.* [68]	Mild to moderate essential hypertensive patients (*n* = 12, 7 males)	Amlodipine (5 mg, 10 mg)	Open label, randomised, crossover study, Morning (0800 h)/evening (2000 h) (3 weeks)	Morning/evening
Mengden *et al.* [69]	Mild to moderate hypertensive patients (*n* = 20)	Amlodipine (5 mg)	Randomised, placebo-controlled open-label, crossover study Morning/evening (8 weeks)	Morning/evening
Qui *et al.* [70]	Mild to moderate essential hypertensive patients (*n* = 60, 44 males) Age: 21–77	Amlodipine (5 mg)	Prospective, double-blind, randomised, crossover study, Morning (0700 h)/evening (2100 h) (12 weeks)	Morning
Zeng *et al.* [63]	Essential hypertensive (*n* = 80), Age: 67 ± 9.8	Amlodipine and hydrochlorothiazide (5 and 25 mg)	Multicentre, open label randomized study Morning (0800 h)/bedtime (2200 h) (12 weeks)	Bedtime
Hoshino *et al.* [64]	Essential hypertensive patients (*n* = 31, 12 males) Age: 69 ± 11	Amlodipine and olmesartan combination (2.5–10 mg and 20–40 mg)	Open-label randomised crossover study Morning/bedtime (32 weeks)	Bedtime
Hermida *et al.* [65]	Untreated uncomplicated essential hypertensive patients (*n* = 203, 92 males) Age: 56.7 ± 12.5	Valsartan (V) and amlodipine (A) (160 mg (V) and 5 mg (A)/day) (medications taken single or together)	PROBE and parallel group study Morning/bedtime (12 weeks)	Bedtime
Asmar *et al.* [71]	Essential hypertensive patients with BP uncontrolled by 5 mg amlodipine (I = 463, 291 males) Age: 56 ± 10	Amlodipine (A)/valsartan (V) combination (5 mg (A) and 160 mg) (increase to 10 mg and 160 mg after 4 weeks)	PROBE, Multicentre, study Morning/evening admin in 2 (12 weeks)	Morning/evening
Kasiskogias *et al.* [66]	Untreated essential hypertensive patients with obstructive sleep apnoea (*n* = 41, 32 males) Age: >30	Amlodipine (A)/valsartan (V) combination (5/160 mg), (10/160 mg), (10/360 mg) or valsartan (160 mg)	Prospective, open label crossover study Morning/evening (16 weeks)	Evening
Shiga *et al.* [72]	Essential hypertensive patients (*n* = 13, 8 males) Age: 46.5 ± 8.4	Atenolol (50 mg)	Randomised, crossover comparative study day trial (0900 h)/night trial (2100 h) (16 days)	Morning/nighttime

PROBE: prospective randomized open-label blinded endpoint.

**Table 4:** Evidence supporting the chronotherapy of proton pump inhibitors.

Reference	Participants (Sample Size) Age in Years	Medicine (Dose)	Study Design (Study Duration)	Suggested Time
Pehlivanov *et al.* [74]	GERD patients (*n* = 20, 6 males) Age: 45.4 ± 9.2	Rabeprazole (20 mg)	Randomised, double-blinded study Morning/evening (7 days)	Evening
Hendel *et al.* [75]	GERD patients (*n* = 17, 7 males) Age: 22–67	Omeprazole (40 mg)	Crossover study Morning (0800–1000 h)/evening (2100–2300 h) (28 days)	Morning/evening

GERD: gastroesophageal reflux disease.

**Table 5:** Evidence supporting the chronotherapy for selected medicines.

Reference	Participants (Sample Size) Age in Years	Medicine (Dose)	Study Design (Study Duration)	Suggested Time
Calverley *et al.* [77]	COPD patients (*n* = 121, 71 males) Age: 65.8 ± 7.9	Tiotropium (18 mg) dry powder device (HandiHaler)	Randomised, double-blind, placebo-controlled Morning (0900 h)/evening (2100 h) before meals (6 weeks)	Morning/evening

## References

[1] Kaur G., Gan Y.-L., Phillips C., Wong K., Saini B. (2016). Chronotherapy in practice: The perspective of the community pharmacist. Int. J. Clin. Pharm..

[2] Lemmer B. (1996). The clinical relevance of chronopharmacology in therapeutics. Pharmacol. Res..

[3] Lemmer B. (2005). Chronopharmacology and controlled drug release. Expert Opin. Drug Deliv..

[4] Lemmer B. (2012). The importance of biological rhythms in drug treatment of hypertension and sex-dependent modifications. Chronophysiol. Ther..

[5] Schulz P., Steimer T. (2009). Neurobiology of circadian systems. CNS Drugs.

[6] Buijs R.M., la Fleur S.E., Wortel J., Van Heyningen C., Zuiddam L., Mettenleiter T.C., Kalsbeek A., Nagai K., Niijima A. (2003). The suprachiasmatic nucleus balances sympathetic and parasympathetic output to peripheral organs through separate preautonomic neurons. J. Comp. Neurol..

[7] Willich S.N., Linderer T., Wegscheider K., Leizorovicz A., Alamercery I., Schröder R. (1989). Increased morning incidence of myocardial infarction in the ISAM study: Absence with prior beta-adrenergic blockade. ISAM study group. Circulation.

[8] Smolensky M.H., Lemmer B., Reinberg A.E. (2007). Chronobiology and chronotherapy of allergic rhinitis and bronchial asthma. Adv. Drug Deliv. Rev..

[9] Smolensky M.H., Reinberg A., Labrecque G. (1995). Twenty-four hour pattern in symptom intensity of viral and allergic rhinitis: Treatment implications. J. Allergy Clin. Immunol..

[10] Mormont M.C., Lévi F. (1997). Circadian-system alterations during cancer processes: A review. Int. J. Cancer.

[11] Quyyumi A.A. (1990). Circadian rhythms in cardiovascular disease. Am. Heart J..

[12] Bellamy N., Sothern R.B., Campbell J., Buchanan W.W. (2002). Rhythmic variations in pain, stiffness, and manual dexterity in hand osteoarthritis. Ann. Rheum. Dis..

[13] Hart F.D., Taylor R.T., Huskisson E.C. (1970). Pain at night. Lancet.

[14] Smolensky M.H., Portaluppi F., Manfredini R., Hermida R.C., Tiseo R., Sackett-Lundeen L.L., Haus E.L. (2015). Diurnal and twenty-four hour patterning of human diseases: Cardiac, vascular, and respiratory diseases, conditions, and syndromes. Sleep Med. Rev..

[15] Smolensky M.H., Portaluppi F., Manfredini R., Hermida R.C., Tiseo R., Sackett-Lundeen L.L., Haus E.L. (2015). Diurnal and twenty-four hour patterning of human diseases: Acute and chronic common and uncommon medical conditions. Sleep Med. Rev..

[16] Ohdo S. (2007). Chronopharmacology focused on biological clock. Drug Metab. Pharmacokinet..

[17] Smolensky M.H., Peppas N.A. (2007). Chronobiology, drug delivery, and chronotherapeutics. Adv. Drug Deliv. Rev..

[18] Smolensky M.H., Siegel R.A., Haus E., Hermida R., Portaluppi F., Siepmann J., Siegel R.A., Rathbone M.J. (2012). Biological rhythms, drug delivery, and chronotherapeutics. Fundamentals and Applications of Controlled Release Drug Delivery.

[19] Ohdo S. (2003). Changes in toxicity and effectiveness with timing of drug administration: Implications for drug safety. Drug Saf..

[20] Erkekoglu P., Baydar T. (2012). Chronopharmacodynamics of drugs in toxicological aspects: A short review for clinical pharmacists and pharmacy practitioners. J. Res. Pharm. Pract..

[21] Erkekoglu P., Baydar T. (2012). Chronopharmacokinetics of drugs in toxicological aspects: A short review for pharmacy practitioners. J. Res. Pharm. Pract..

[22] Bruguerolle B., Boulamery A., Simon N. (2008). Biological rhythms: A neglected factor of variability in pharmacokinetic studies. J. Pharm. Sci..

[23] Lemmer B., Bruguerolle B. (1994). Chronopharmacokinetics—Are they clinically relevant?. Clin. Pharmacokinet..

[24] Bruguerolle B. (1998). Chronopharmacokinetics. Current status. Clin. Pharmacokinet..

[25] Paschos G.K., Baggs J.E., Hogenesch J.B., FitzGerald G.A. (2010). The role of clock genes in pharmacology. Annu. Rev. Pharmacol. Toxicol..

[26] Lemmer B., Nold G. (1991). Circadian changes in estimated hepatic blood flow in healthy subjects. Br. J. Clin. Pharmacol..

[27] Reinberg A., Smolensky M., Labrecque G. (2013). Annual Review of Chronopharmacology.

[28] Ohdo S. (2010). Chronotherapeutic strategy: Rhythm monitoring, manipulation and disruption. Adv. Drug Deliv. Rev..

[29] Ohdo S., Wang D.S., Koyanagi S., Takane H., Inoue K., Aramaki H., Yukawa E., Higuchi S. (2000). Basis for dosing time-dependent changes in the antiviral activity of interferon-alpha in mice. J. Pharmacol. Exp. Ther..

[30] Nainwal N. (2012). Chronotherapeutics—A chronopharmaceutical approach to drug delivery in the treatment of asthma. J. Control. Release.

[31] Lemmer B. (2007). Chronobiology and chronopharmacology of hypertension. Blood Pressure Monitoring in Cardiovascular Medicine and Therapeutics.

[32] Hermida R.C., Ayala D.E., Fernández J.R., Portaluppi F., Fabbian F., Smolensky M.H. (2011). Circadian rhythms in blood pressure regulation and optimization of hypertension treatment with ACE inhibitor and ARB medications. Am. J. Hypertens..

[33] Smolensky M.H., Hermida R.C., Ayala D.E., Tiseo R., Portaluppi F. (2010). Administration-time-dependent effects of blood pressure-lowering medications: Basis for the chronotherapy of hypertension. Blood Press. Monit..

[34] Smolensky M.H., Hermida R.C., Ayala D.E., Portaluppi F. (2015). Bedtime hypertension chronotherapy: Concepts and patient outcomes. Curr. Pharm. Des..

[35] Lévi F., Focan C., Karaboué A., de la Valette V., Focan-Henrard D., Baron B., Kreutz F., Giacchetti S. (2007). Implications of circadian clocks for the rhythmic delivery of cancer therapeutics. Adv. Drug Deliv. Rev..

[36] Bruguerolle B., Labrecque G. (2007). Rhythmic pattern in pain and their chronotherapy. Adv. Drug Deliv. Rev..

[37] Piper M.A., Evans C.V., Burda B.U., Margolis K.L., O’Connor E., Whitlock E.P. (2015). Diagnostic and predictive accuracy of blood pressure screening methods with consideration of rescreening intervals: A systematic review for the US preventive services task force. Ann. Intern. Med..

[38] Head G.A., McGrath B.P., Mihailidou A.S., Nelson M.R., Schlaich M.P., Stowasser M., Mangoni A.A., Cowley D., Brown M.A., Ruta L.-A. (2012). Ambulatory blood pressure monitoring in Australia: 2011 consensus position statement. J. Hypertens..

[39] Smolensky M.H. (2002). Compliance to prescription medications entails respect for treatment timing. Chronobiol. Int..

[40] Medicare Pharmaceutical Benefits Scheme. http://www.medicareaustralia.gov.au/provider/pbs/.

[41] Expenditure and Prescriptions Twelve Months to 30 June 2014. http://www.pbs.gov.au/info/statistics/expenditure-and-prescriptions-30-06-2014.

[42] WHO Model Lists of Essential Medicines. http://www.who.int/medicines/publications/essentialmedicines/en/index.html.

[43] Bulsara C.E., McKenzie A. (2009). The quality of medication information in Australia: The need for more clinical expertise and accountability. Med. J. Aust..

[44] Koo M.M., Krass I., Aslani P. (2003). Factors influencing consumer use of written drug information. Ann. Pharmacother..

[45] Williams I.D. (2007). Should clinical software be regulated?. Med. J. Aust..

[46] Raymond C., Cho L., Rocco M., Hazen S.L. (2014). New guidelines for reduction of blood cholesterol: Was it worth the wait?. Clevel. Clin. J. Med..

[47] Boggia J., Li Y., Thijs L., Hansen T.W., Kikuya M., Björklund-Bodegård K., Richart T., Ohkubo T., Kuznetsova T., Torp-Pedersen C. (2007). Prognostic accuracy of day versus night ambulatory blood pressure: A cohort study. Lancet.

[48] Ozaydin M., Dede O., Dogan A., Aslan S.M., Altinbas A., Ozturk M., Varol E., Turker Y. (2006). Effects of morning versus evening intake of atorvastatin on major cardiac event and restenosis rates in patients undergoing first elective percutaneous coronary intervention. Am. J. Cardiol..

[49] Saito Y., Yoshida S., Nakaya N., Hata Y., Goto Y. (1991). Comparison between morning and evening doses of simvastatin in hyperlipidemic subjects. A double-blind comparative study. Arterioscler. Thromb. Vasc. Biol..

[50] Wallace A., Chinn D., Rubin G. (2003). Taking simvastatin in the morning compared with in the evening: Randomised controlled trial. BMJ.

[51] Lund T.M., Torsvik H., Falch D., Christophersen B., Skardal R., Gullestad L. (2002). Effect of morning versus evening intake of simvastatin on the serum cholesterol level in patients with coronary artery disease. Am. J. Cardiol..

[52] Tharavanij T., Wongtanakarn S., Lerdvuthisopon N., Teeraaunkul S., Youngsriphithak P., Sritipsukho P. (2010). Lipid lowering efficacy between morning and evening simvastatin treatment: A randomized double-blind study. J. Med. Assoc. Thai..

[53] Plakogiannis R., Cohen H., Taft D. (2005). Effects of morning versus evening administration of atorvastatin in patients with hyperlipidemia. Am. J. Health Syst. Pharm..

[54] Kim S.H., Kim M.K., Seo H.S., Hyun M.S., Han K.R., Cho S.W., Kim Y.K., Park S.H. (2013). Efficacy and safety of morning versus evening dose of controlled-release simvastatin tablets in patients with hyperlipidemia: A randomized, double-blind, multicenter phase III trial. Clin. Ther..

[55] Yi Y.J., Kim H.J., Jo S.K., Kim S.G., Song Y.R., Chung W., Han K.H., Lee C.H., Hwang Y.H., Oh K.H. (2014). Comparison of the efficacy and safety profile of morning administration of controlled-release simvastatin versus evening administration of immediate-release simvastatin in chronic kidney disease patients with dyslipidemia. Clin. Ther..

[56] Yoon H.S., Kim S.H., Kim J.K., Ko S.H., Ko J.E., Park S.J., Park M.G., Lee J.H., Hyon M.S. (2011). Comparison of effects of morning versus evening administration of ezetimibe/simvastatin on serum cholesterol in patients with primary hypercholesterolemia. Ann. Pharmacother..

[57] Plakogiannis R., Cohen H. (2007). Optimal low-density lipoprotein cholesterol lowering—Morning versus evening statin administration. Ann. Pharmacother..

[58] Morgan T., Anderson A., Jones E. (1997). The effect on 24 h blood pressure control of an angiotensin converting enzyme inhibitor (perindopril) administered in the morning or at night. J. Hypertens..

[59] Hermida R.C., Ayala D.E. (2009). Chronotherapy with the angiotensin-converting enzyme inhibitor ramipril in essential hypertension: Improved blood pressure control with bedtime dosing. Hypertension.

[60] Hermida R.C., Ayala D.E., Fernández J.R., Calvo C. (2007). Comparison of the efficacy of morning versus evening administration of telmisartan in essential hypertension. Hypertension.

[61] Pechère-Bertschi A., Nussberger J., Decosterd L., Armagnac C., Sissmann J., Bouroudian M., Brunner H.R., Burnier M. (1998). Renal response to the angiotensin II receptor subtype 1 antagonist irbesartan versus enalapril in hypertensive patients. J. Hypertens..

[62] Eguchi K., Shimizu M., Hoshide S., Shimada K., Kario K. (2012). A bedtime dose of ARB was better than a morning dose in improving baroreflex sensitivity and urinary albumin excretion—The J-TOP study. Clin. Exp. Hypertens..

[63] Zeng J., Jia M., Ran H., Tang H., Zhang Y., Zhang J., Wang X., Wang H., Yang C., Zeng C. (2011). Fixed-combination of amlodipine and diuretic chronotherapy in the treatment of essential hypertension: Improved blood pressure control with bedtime dosing—A multicenter, open-label randomized study. Hypertens. Res..

[64] Hoshino A., Nakamura T., Matsubara H. (2010). The bedtime administration ameliorates blood pressure variability and reduces urinary albumin excretion in amlodipine-olmesartan combination therapy. Clin. Exp. Hypertens..

[65] Hermida R.C., Ayala D.E., Fontao M.J., Mojón A., Fernández J.R. (2010). Chronotherapy with valsartan/amlodipine fixed combination: Improved blood pressure control of essential hypertension with bedtime dosing. Chronobiol. Int..

[66] Kasiakogias A., Tsioufis C., Thomopoulos C., Andrikou I., Aragiannis D., Dimitriadis K., Tsiachris D., Bilo G., Sideris S., Filis K. (2015). Evening versus morning dosing of antihypertensive drugs in hypertensive patients with sleep apnoea: A cross-over study. J. Hypertens..

[67] Myburgh D.P., Verho M., Botes J.H., Erasmus T.P., Luus H.G. (1995). 24-hour blood pressure control with ramipril: Comparison of once-daily morning and evening administration. Curr. Ther. Res. Clin. Exp..

[68] Nold G., Strobel G., Lemmer B. (1998). Morning versus evening amlodipine treatment: Effect on circadian blood pressure profile in essential hypertensive patients. Blood Press. Monit..

[69] Mengden T., Binswanger B., Spuhler T., Weisser B., Vetter W. (1993). The use of self-measured blood pressure determinations in assessing dynamics of drug compliance in a study with amlodipine once a day, morning versus evening. J. Hypertens..

[70] Qiu Y.G., Chen J.Z., Zhu J.H., Yao X.Y. (2003). Differential effects of morning or evening dosing of amlodipine on circadian blood pressure and heart rate. Cardiovasc. Drugs Ther..

[71] Asmar R., Gosse P., Quere S., Achouba A. (2011). Efficacy of morning and evening dosing of amlodipine/valsartan combination in hypertensive patients uncontrolled by 5 mg of amlodipine. Blood Press. Monit..

[72] Shiga T., Fujimura A., Tateishi T., Ohashi K., Ebihara A. (1993). Differences of chronopharmacokinetic profiles between propranolol and atenolol in hypertensive subjects. J. Clin. Pharmacol..

[73] American Diabetes Association (2014). Standards of medical care in diabetes—2014. Diabetes Care.

[74] Pehlivanov N.D., Olyaee M., Sarosiek I., McCallum R.W. (2003). Comparison of morning and evening administration of rabeprazole for gastro-oesophageal reflux and nocturnal gastric acid breakthrough in patients with reflux disease: A double-blind, cross-over study. Aliment. Pharmacol. Ther..

[75] Hendel J., Hendel L., Aggestrup S. (1995). Morning or evening dosage of omeprazole for gastro-oesophageal reflux disease?. Aliment. Pharmacol. Ther..

[76] Highlights of prescribing information—ACIPHEX. https://www.accessdata.fda.gov/drugsatfda_docs/label/2014/020973s035204736s005lbl.pdf.

[77] Calverley P.M.A., Lee A., Towse L., van Noord J., Witek T.J., Kelsen S. (2003). Effect of tiotropium bromide on circadian variation in airflow limitation in chronic obstructive pulmonary disease. Thorax.

[78] Enas E.A., Kuruvila A., Khanna P., Pitchumoni C.S., Mohan V. (2013). Benefits & risks of statin therapy for primary prevention of cardiovascular disease in Asian Indians—A population with the highest risk of premature coronary artery disease & diabetes. Indian J. Med. Res..

[79] Singh R., Sharma P.K., Malviya R. (2010). Review on chronotherapeutics—A new remedy in the treatment of various diseases. Eur. J. Biol. Sci..

[80] Zhu L.L., Zhou Q., Yan X.F., Zeng S. (2008). Optimal time to take once-daily oral medications in clinical practice. Int. J. Clin. Pract..

[81] Liao J.K. (2002). Isoprenoids as mediators of the biological effects of statins. J. Clin. Investig..

[82] Jellinger P., Smith D., Mehta A., Ganda O., Handelsman Y., Rodbard H., Shepherd M., Seibel J. (2012). American association of clinical endocrinologists’ guidelines for management of dyslipidemia and prevention of atherosclerosis. Endocr. Pract..

[83] Lemmer B. (1991). Circadian rhythms and drug delivery. J. Control. Release.

[84] Lemmer B. (2000). Relevance for chronopharmacology in practical medicine. Semin. Perinatol..

[85] Hermida R.C., Smolensky M.H., Ayala D.E., Fernández J.R., Moyá A., Crespo J.J., Mojón A., Ríos M.T., Fabbian F., Portaluppi F. (2014). Abnormalities in chronic kidney disease of ambulatory blood pressure 24 h patterning and normalization by bedtime hypertension chronotherapy. Nephrol. Dial. Transplant..

[86] Wang C., Deng W.J., Gong W.Y., Zhang J., Tang H., Peng H., Zhang Q.Z., Ye Z.C., Lou T. (2015). High prevalence of isolated nocturnal hypertension in chinese patients with chronic kidney disease. J. Am. Heart Assoc..

[87] Hansen T.W., Li Y., Boggia J., Thijs L., Richart T., Staessen J.A. (2011). Predictive role of the nighttime blood pressure. Hypertension.

[88] Yusuf S., Sleight P., Pogue J., Bosch J., Davies R., Dagenais G. (2000). Effects of an angiotensin-converting-enzyme inhibitor, ramipril, on cardiovascular events in high-risk patients. The heart outcomes prevention evaluation study investigators. N. Engl. J. Med..

[89] Svensson P., de Faire U., Sleight P., Yusuf S., Östergren J. (2001). Comparative effects of ramipril on ambulatory and office blood pressures a HOPE substudy. Hypertension.

[90] Hermida R.C., Ayala D.E., Mojón A., Fernández J.R. (2010). Influence of circadian time of hypertension treatment on cardiovascular risk: Results of the MAPEC study. Chronobiol. Int..

[91] Stranges P.M., Drew A.M., Rafferty P., Shuster J.E., Brooks A.D. (2015). Treatment of hypertension with chronotherapy: Is it time of drug administration?. Ann. Pharmacother..

[92] Gibaldi M. (1991). Biopharmaceutics and Clinical Pharmacokinetics.

[93] Koopman M.G., Koomen G.C., Krediet R.T., de Moor E.A., Hoek F.J., Arisz L. (1989). Circadian rhythm of glomerular filtration rate in normal individuals. Clin. Sci..

[94] Khan Z., Pillay V., Choonara Y.E., du Toit L.C. (2009). Drug delivery technologies for chronotherapeutic applications. Pharm. Dev. Technol..

[95] Vaughn B.V., Rotolo S., Roth H.L. (2014). Circadian rhythm and sleep influences on digestive physiology and disorders. Chronophysiol. Ther..

[96] Chokroverty S. (2013). Sleep Disorders Medicine: Basic Science, Technical Considerations, and Clinical Aspects.

[97] Stockigt J.R. (2007). Barriers in the quest for quality drug information: Salutary lessons from TGA-approved sources for thyroid-related medications. Med. J. Aust..

[98] Fujimura A. (2013). [Chronotherapy--present and future]. Nihon Rinsho.

[99] Durrington H.J., Farrow S., Ray D. (2014). Recent advances in chronotherapy for the management of asthma. Chronophysiol. Ther..

[100] Hassan A., Haefeli W.E. (2010). Appropriateness of timing of drug administration in electronic prescriptions. Pharm. World Sci..

[101] Smolensky M.H. (1998). Knowledge and attitudes of American physicians and public about medical chronobiology and chronotherapeutics. Findings of two 1996 gallup surveys. Chronobiol. Int..

[102] Kaur G., Saba M., Phillips C.L., Wong K., Saini B. (2015). Education intervention on chronotherapy for final-year pharmacy students. Pharmacy.

[103] De Giorgi A., Menegatti A.M., Fabbian F., Portaluppi F., Manfredini R. (2013). Circadian rhythms and medical diseases: Does it matter when drugs are taken?. Eur. J. Intern. Med..

